# Optimization and Modelling the Mechanical Performance of Date Palm Fiber-Reinforced Concrete Incorporating Powdered Activation Carbon Using Response Surface Methodology

**DOI:** 10.3390/ma16082977

**Published:** 2023-04-08

**Authors:** Musa Adamu, Yasser E. Ibrahim, Mahmoud M. Abdel daiem, Hani Alanazi, Oussama Elalaoui, Nageh M. Ali

**Affiliations:** 1 Engineering Management Department, College of Engineering, Prince Sultan University, Riyadh 11586, Saudi Arabia; madamu@psu.edu.sa (M.A.); ymansour@psu.edu.sa (Y.E.I.); 2 Environmental Engineering Department, Faculty of Engineering, Zagazig University, Zagazig 44519, Egypt; mmabdeldaiem@eng.zu.edu.eg; 3 Department of Civil Engineering, College of Engineering, Shaqra University, Al-Dawadmi 11911, Saudi Arabia; 4 Department of Civil and Environmental Engineering, College of Engineering, Majmaah University, Al-Majmaah 11952, Saudi Arabia; o.elalaoui@mu.edu.sa (O.E.); nageh82@mu.edu.sa (N.M.A.); 5 Department of Civil Engineering, College of Engineering, Assiut University, Assiut 71511, Egypt

**Keywords:** date palm fiber, natural fiber, powdered activated carbon, mechanical properties, response surface methodology

## Abstract

Date palm fiber (DPF) has been reported to have many advantages when used in concrete, however, its major disadvantage is that it causes a reduction in compressive strength. In this research, powdered activated carbon (PAC) was added to cement in the DPF-reinforced concrete (DPFRC) to lessen the loss in strength. PAC has not been properly utilized as an additive in fiber reinforced concrete even though it has been reported to enhance the properties of cementitious composites. Response surface methodology (RSM) has also been utilized for experimental design, model development, results analysis, and optimization. The variables were DPF and PAC as additions each at proportions of 0%, 1%, 2%, and 3% by weight of cement. Slump, fresh density, mechanical strengths, and water absorption were the responses that were considered. From the results, both DPF and PAC decreased the workability of the concrete. DPF addition improved the splitting tensile and flexural strengths and reduced the compressive strength, and up to 2 wt% PAC addition enhanced the concrete’s strength and lowered the water absorption. The proposed models using RSM were extremely significant and have excellent predictive power for the concrete’s aforementioned properties. Each of the models was further validated experimentally and was found to have an average error of less than 5.5%. According to the results of the optimization, the optimal mix of 0.93 wt% DPF and 0.37 wt% PAC as cement additives resulted in the best properties of the DPFRC in terms of workability, strength, and water absorption. The optimization’s outcome received a 91% desirability rating. The addition of 1% PAC increased the 28-day compressive strength of the DPFRC containing 0%, 1% and 2% DPF by 9.67%, 11.13% and 5.5% respectively. Similarly, 1% PAC addition enhanced the 28-day split tensile strength of the DPFRC containing 0%, 1% and 2% by 8.54%, 11.08% and 19.3% respectively. Likewise, the 28-day flexural strength of DPFRC containing 0%, 1%, 2% and 3% improved by 8.3%, 11.15%, 18.7% and 6.73% respectively with the addition of 1% PAC. Lastly, 1% PAC addition led to a reduction in the water absorption of DPFRC containing 0% and 1% DPF by 17.93% and 12.2% respectively.

## 1. Introduction

One of the best ways of achieving sustainability is by proper utilization of waste materials and natural resources mostly in the building and construction sectors. This is because the construction industry is a major consumer of many natural resources. Being the most widely utilized and second-most utilized substance after water, concrete requires enormous amounts of material to make [1,2,3]. When exposed to tensile or bending stress, concrete easily cracks due to its poor tensile strength and strain characteristics, but it has excellent compressive strength. The construction industry addresses this shortcoming of concrete through the use of reinforcement to form a composite structure called reinforced concrete. Steel reinforcement is an expensive material, and this increases the cost of buildings and construction. Hence, innovations to boost the tensile and strain characteristics of the concrete were derived by several researchers by using admixtures, fibers, and supplementary cementitious materials to the concrete. This will decrease the quantity of reinforcement needed to generate a reinforced concrete structure and hence reduce the overall cost of the construction [2,4]. Fibers are added to cementitious composites as the main or secondary reinforcement. The fibers are employed to increase the material’s flexural and tensile strength, toughness, and impact resistance as well as to stop cracks from occurring and propagating. When used as secondary reinforcement, fibers are added to control crack formation and its propagation induced by temperature changes or humidity, and also to increase the post-cracking load resistance caused by spalling or overload [5,6]. Additionally, fibers have been found to enhance the heat resistance of cementitious composites by reducing the risk of thermal spalling. For example, steel fiber, due to its high melting point, can act on the thermal stress mechanism and prevent thermal cracks from propagating, whereas polypropylene fiber, which has a low melting point, can act on the pore pressure mechanism [7].

Natural fibers are acquired from natural resources such as agricultural products or animals. They are mostly characterized by high tensile strength and low elastic modulus [8]. Natural fibers have a series of advantages over synthetic fibers, which includes availability, lower cost and density, less abrasiveness, higher acoustic and thermal insulation properties, and high resistance to the alkaline environment [9,10]. However, natural fibers possess some disadvantages compared to synthetic fibers, which include high water absorption, poor wettability, and high hydroxyl (OH) content which instigates hydrophilicity to the fiber, causing poor fiber-to-fiber bonding and cement matrix, and thermal and mechanical degradation [5]. To address these shortcomings of natural fibers, researchers have utilized some methods including graft copolymerization and treatment of the fiber in an alkaline or saline environment. However, alkaline therapy was found to be the most efficient method [11,12,13].

DPF is among the natural fibers obtained from the date palm tree, and is readily available globally, especially in north African countries and the Middle East [14]. DPF has been utilized in cement composites, such as mortar and concrete, as a natural fiber. According to previous studies, DPF-reinforced cementitious composites provide several benefits over traditional cementitious composites, including reduced density and lighter weight, better thermal insulation, improved acoustic characteristics, and lower thermal conductivity [15,16]. Furthermore, it has been noted that DPF can lower the spread of cracks in cementitious composite and increase ductility, energy absorption capacity, and tensile and flexural strength [4,5,17,18]. Cementitious composites’ compressive strength has indeed been found to decline due to the inclusion of DPF. The air entrapment from the DPF during mixing and casting increases porosity and poor gluing between the cement matrix and DPF. This is because of the high hydrophilic nature and high absorption of the DPF, which causes a reduction in consistency and results in honeycomb and porosity in the hardened cement matrix, and difficult compaction and packing of the fresh concrete mix containing the DPF. As a result, this leads to a drop in the compressive strength of the composites with regards to DPF addition [4,15]. Numerous investigations have been done to decrease the devastating impact of DPF. Some of the methods include treatment of the DPF using alkaline solutions such as NaOH and Ca(OH)_2_ [5,19]. Another effective method is adding SCM such as silica fume to the DPF concrete [4]. Other additives such as slag and fly ash have reportedly been utilized in fiber-reinforced concrete to reduce chemical interactions among the cement matrix and fibers and to strengthen the link within the fiber-matrix interphase [5,6].

An activated carbon (AC) is a carbon-based material obtained from carbonaceous materials such as wood, coir, coconut husk, rick husk, coal, petroleum pitch, and lignite. The AC is produced from either a chemical or a physical activation process. There are several types of AC, which include granular, extruded, bead, powdered, polymer-coated, and impregnated ACs. Powdered activated carbon (PAC) is produced by grinding granular AC; it has a wide surface area and is very porous to be able to absorb chemical components such as mercury [20,21]. AC was used as a cement additive in industrial applications dating back to 1952 for the prevention of the contamination of oil well linings when in contact with drilling muds [22,23]. AC in powdered and granular form was used for different engineering applications which included; (a) as an additive to cementitious materials such as concrete for the absorption of nitrogen oxides (NOx) and other volatile organic compounds from combustion in parking garages and roadway tunnels; (b) as aggregate for making lightweight concrete; (c) for preventing microbial-induced corrosion in concrete sewer pipelines, (d) in self-healing concrete for the conveyance of bacteria to aid the self-healing process [23,24,25,26,27,28]. PACs, due to their high porosity at nano scale and larger surface area, make good materials for the densification of microstructure at nano levels [29]. PAC has been used as an additive in concrete and mortar. Na, et al. [30] observed up to 1.5% PAC as additive enhanced the strength of mortar. Additionally, they testified that the inclusion of PAC improved the strength-hardening process, which decreased the curing time. Zheng, et al. [31] reported an enhancement in fly ash mortar’s compressive strength with the addition of PAC. From the microstructural analysis, they observed that PAC acted as a filler and densified the mortar microstructure, hence improving the strengths. Wang, et al. [32] added PAC at a proportion of 0.5% to 2.0% by weight of binder in mortar with and without fly ash. They reported that the PAC had a very good dispersing ability in the cement matrix. Due to the filler action of PAC, the pore volume of the mortar, whether it contained fly ash or not, decreased when it was added.

DPF has been reported to have many positive advantages when used in composites. However, the main setback of utilizing DPF in mortar and concrete is escalation in porosity and diminution in strengths of the composites. The negative impacts of the DPF in mortar and concrete have been reduced using a variety of techniques, some of which were unsuccessful and others which were successful but expensive. Because PAC is a less expensive material, it has been shown to increase the mechanical strengths of mortar and concrete by densifying the microstructure and pore volume. There are scanty or no available studies that utilized PAC in DPF-reinforced concrete in order to lessen the negative impacts of the DPF. This research used PAC as an additive to weight for cementitious material in DPF-reinforced concrete. Response surface methodology (RSM) was used to predict the properties of the composite, investigate the impact of PAC and DPF on the concrete’s characteristics, and design the experiments.

## 2. Materials and Methods

### 2.1. Materials

**Figure 1:**
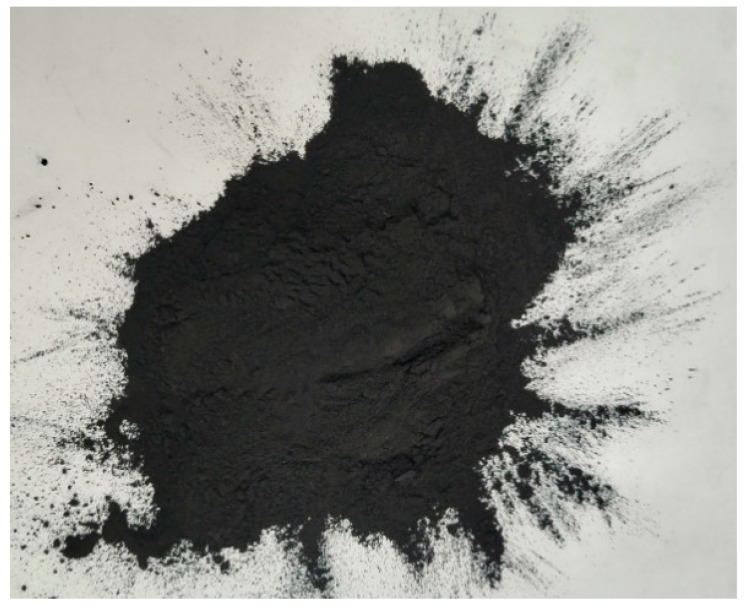
Activated carbon in powdered form.

Ordinary Portland Type I cement served as the major binder material in this research. The cement has a specific gravity of 3.15 and bulk density of 1440 kg/m^3^. The chemical properties of the cement highlighted in Table 1 were obtained through an X-Ray Fluorescence (XRF) Spectrometer test, revealing that it conformed with the standard specifications of ASTM C150/150M [33]. Commercially available activated carbon in powdered form obtained from BMS factory Saudi Arabia, as shown in Figure 1, was used as additive to cement. The PAC possesses a specific surface area larger than 3000 m^2^/g, iodine number of 1450 mg/g, bulk density of 0.55 g/cm^3^, and a mean pore diameter of 2.14 nm. The properties of the PAC as obtained through XRF analysis are also depicted in Table 1.

Natural river sand was utilized as the fine aggregate. The physical properties of the fine aggregate are summarized in Table 2. For coarse aggregate, 19 mm-sized crushed gravel was utilized. Also, for the coarse aggregate, its physical properties are shown in Table 2.

**Figure 2:**
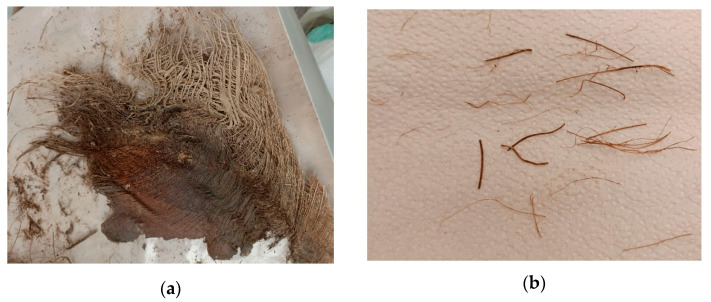
DPF used in the study. (**a**) Raw DPF in interwoven form, (**b**) Treated single DPF [4].

The DPF was sourced from a nearby date palm tree farm in raw form. The fibers were in quadrilateral interwoven mesh forms with approximate length between 300–500 mm and breadth of 200–300 mm as shown in Figure 2a. The raw DPF was first immersed in water for about three hours and then separated into individual fibers of length between 20–30 mm and diameter between 0.2–1.0 mm as shown in Figure 2b. The single DPFs were then immersed in 3% NaOH solution (alkaline solution) to eliminate all the impurities and dirt. The DPFs were then washed thoroughly in clean water after alkaline treatment and dried in air for 48 h to bring it to a completely dry state before being incorporated into the concrete.

### 2.2. Mix Proportioning with RSM

The procedure listed in ACI 211.1R [34] was used as a guidance in designing the control concrete mix. The DPFs were added in various proportions of 0%, 1%, 2%, and 3% by weight of cement to the concrete. Additionally, PAC was incorporated into the cement in different proportions of 0%, 1%, 2%, and 3% by weight of cement.

The response surface methodology (RSM) is an appropriate statistical and mathematical technique used in the design of experimental mixes. It can also be used for the development of mathematical models to predict one or more concrete properties based on one or more input factors (variables) [17,35]. RSM can further be used to determine the optimum mix proportion that will yield the best performance or any desired concrete’s property through optimization (multi-objective) by establishing the desired targets for both the variables and the responses [36,37]. Either Minitab or Design Expert software was utilized for the RSM analysis [36,37].

A globalized model in the form of a linear mathematical equation can be utilized to represent the relationship between the input variables and responses as presented in Equation (1). However, in reality, curvature mostly does exist, hence the linear equation is not best suited for the task. Therefore, the most appropriate relationship between the variables and responses are polynomials of second or higher orders as presented in Equation (2) [36,38].

(1) $$Z=\alpha_{0}+\alpha_{1}T_{1}+\alpha_{2}T_{2}+\ldots {\alpha_{n}T}_{n}+\Psi$$

(2) $$Z=\alpha_{0}+\sum_{i=1}^{n}\alpha_{i}T_{i}+\sum_{i=1}^{n}\alpha_{ii}T_{i}^{2}+\sum_{i<}\sum_{j}\alpha_{ij}T_{i}T_{j}+\Psi$$

in the above equations, Ζ and T designate the response and variables respectively, *α*_0_ is the intercept at which T_1_ = T_2_ = 0, *α* represents the coefficient’s variables, *i* and *j* denote linear and quadratic encrypted quantities respectively, *n* stands for the numeral’s variables, and Ψ denotes an error.

For the RSM analysis and modeling, design expert software (V.11) was utilized. A historical data model was chosen due to its flexibility and simplicity. The input variables include DPF and PAC all as additives by weight of cement. Both the DPF and PAC were added at proportions of 1%, 2% and 3% by weight of cement. The mix proportions were generated based on the aforementioned variables as depicted in Table 3. The slump, fresh density, compressive strength, flexural strength, split tensile strength, and water absorption of the concrete were the responses considered. For the purpose of calculating the lack of fits in the models, which is one of the measures for checking the statistical fitness of the RSM models, the central mix, i.e., mixes with 2% DPF and 2% PAC (M8), were repeated five times. Mix M1 in Table 3 represents the control mix with 0% DPF and 0% PAC [17].

### 2.3. Sample Preparations and Experimental Methods

**Figure 3:**
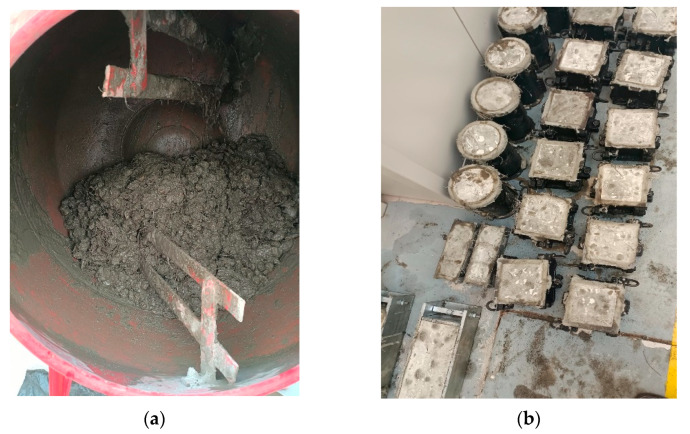
Samples preparations. (**a**) Mixing, (**b**) Hardened samples [4].

The proposed mixes in Table 3, i.e., 13 mixes, were produced in the laboratory and subjected to the desired tests. Concrete sampling, mixing, batching, and curing were all carried out in accordance with ASTM C192/C192M [39]. The mixing of the fresh concrete was carried out in the laboratory using a rotating pan mixer. All the constituent materials were weighed based on the mix proportions. The cement fine aggregate and PAC were firstly poured into the mixer and mixed thoroughly for about 60 s. The water was mixed with superplasticizer before adding. The coarse aggregate was added to the mixer and the mixing continued. The DPF and mixing water were added gently to avoid agglomeration of the fiber. After all the constituent materials were completely added, the mixing continued until a homogenous paste was formed, and all fibers were dispersed. Figure 3a shows the freshly mixed concrete inside the rotating drum mixer. Immediately after mixing, the workability using a slump test and the fresh density of the concrete were determined. The fresh concrete was then cast into the designated molds. Prior to casting, the molds were tightened and oiled for ease of demolding. The fresh concrete in the molds were then kept for 24 h in the laboratory to harden. Figure 3b shows the hardened concrete samples before demolding. After demolding, the samples were placed in a curing tank full of clean water until the recommended testing date.

### 2.4. Experimental Methods

The slump test was used to measure the fresh concrete’s workability immediately after mixing. The ASTM C143/C143M [40] standard technique was followed for conducting the slump test using the slump cone apparatus. According to the procedures outlined in ASTM C138/C138M [41] the fresh density test was performed on the fresh concrete samples. Cubic molds and weighing balance were utilized for testing the density of the fresh concrete. For both the slump and fresh density test, three samples were tested for each of the mixes and the average results reported.

**Figure 4:**
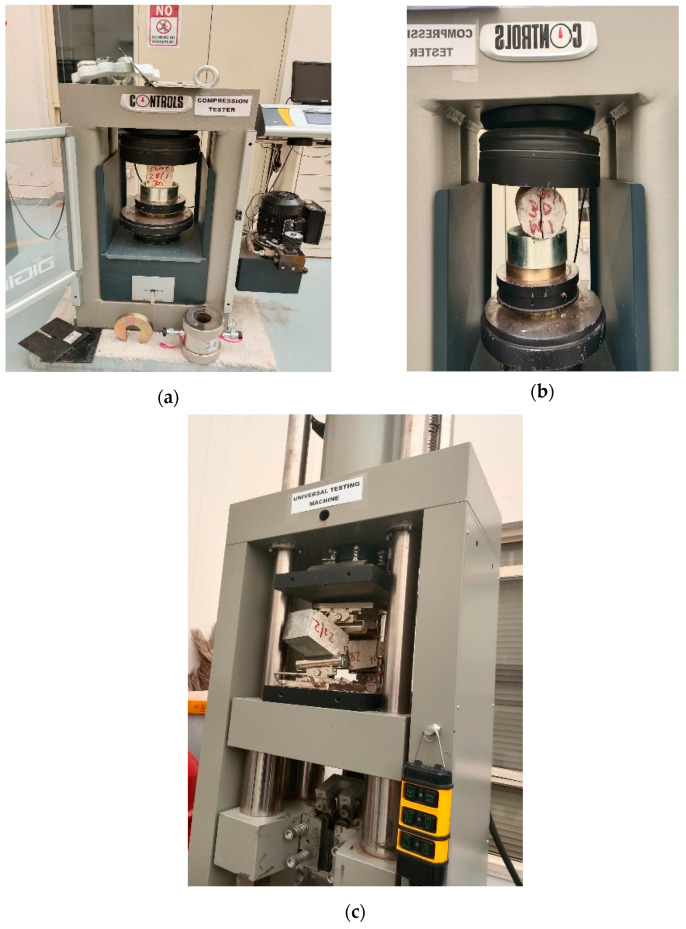
Experimental Methods. (**a**) Compressive Strength Testing, (**b**) Split Tensile Strength Testing, (**c**) Flexural Strength Testing [4].

For the compressive strength test, cubes of 100 mm sizes were produced and subjected to curing in water for 3, 7, and 28 days. The techniques prescribed in BS EN 12390-3 [42] were adopted for the compression testing, with the aid of a universal compression machine of 2000 kN capacity. The experimental set up for the compressive strength is presented in Figure 4a. With reference to the split tensile strength test, cylindric specimens having sizes of 200 mm height and 100 mm diameter were made and cured in water for 3, 7, and 28 days prior to testing. A 2000 kN-capacity universal compression machine was used to measure the tensile strength in accordance with the procedures described in BS EN 12390-6 [43], as shown in Figure 4b. Prismatic samples 100 × 100 × 500 mm size were produced and preserved in water for 7 and 28 days before testing for the flexural strength. The flexural strength test was carried out according to the ASTM C78/C78M [44] specifications, using a beam with a third-point load. A universal testing machine of 3000 kN capacity was utilized for the flexural strengths of the concrete as shown in Figure 4c. For each of the strengths tests, three samples were prepared and tested for each mix and each of the curing periods, and the average results were recorded.

The water absorption test was done using cubic samples of 100 mm diameter after curing in water for 28 days. The test was carried out using the procedures outlined in ASTM C642 [45]. An electric oven, weighing balance and curing tank were the equipment utilized for the water absorption testing. For the water absorption test, three samples were tested for each of the mixes and the mean value recorded.

## 3. Results and Discussion

The experimental results for the DPF-reinforced concrete (DFPRC) mixes are given in Table 4. The results were used for analysis and modelling the properties of the DPFRC by RSM.

### 3.1. Analysis of Variance for RSM Models

#### 3.1.1. Analysis of Variance for Slump and Density

Table 5 displays the results of the analysis of variance (ANOVA) for the models established to estimate the slump and fresh density of the DPF-reinforced concrete (DPFRC) modified with PAC. According to the ANOVA summary, all models for estimating the slump and fresh density of the concrete were statistically significant because their P-scores were lower than 0.05. The letters D and A symbolize DPF and PAC, respectively, in each of the models. Employing a confidence interval (*p* < 0.05), the significance of each term in the models were also examined. Just the terms D and A were significant for the slump model; the other terms were non-significant. The significant terms for the fresh density model were D, A, and A^2^. Additionally, the *p* < 0.05 was used to check the significance of the lack of fit for each model relative to their pure errors. A fitting model must have a non-significant lack of fit. A badly fitted model is indicated by a significant lack of fit [36,37]. The related *p*-values for the lack of fits for the slump and fresh density models were more than 0.05, hence they were not statistically significant. The experimental results were well correlated with the models. For the DPFRC modified with PAC, the models shown as Equations (3) and (4) can be adopted for estimating its slump and fresh density, respectively.

(3) $$Slump=86.8-3.212\times D-9.314\times A+0.359\times D\times A+0.0371\times D^{2}-0.443\times A^{2}$$

(4) $$D_{F}=2457.37-11.971\times D+137.55\times A+0.539\times D\times A-9.20\times D^{2}-54.848\times A^{2}$$

in the above equations, *D_F_* denotes fresh density (kg/m^3^), *D* and *A* denotes DPF, and PAC contents accordingly in %.

The correlation degree (R^2^) was also used to further assess the models’ robustness, fitness, and accuracy. The R^2^ values varied from 0 to 1, with values close to 1 being considered to have high correlation and values near to zero being considered to have low correlation [17,46]. Table 5 displays a summary of the ANOVA for regression coefficient for the designed models. The R^2^ values for the slump and fresh density models were very high. The slump and fresh density models’ R^2^ values of 0.961 and 0.95 indicated that only roughly 3.9% and 5%, respectively, of the entire experimental data were not fully captured by the models. From Table 5, the slump and fresh density models all had variations between their predicted and adjusted R^2^ to be inferior to 0.2. This provided further proof that the models were adequate and suited for purpose. All the models could explore the design space as they had adequate precisions larger than 4 [37,47].

#### 3.1.2. Analysis of Variance for Compressive Strength Models

The established model equations for the prediction of the compressive strengths of the DPFRC, utilizing DPF and PAC as the input variables, are presented in form of quadratic equations in Equations (5) and (6).

(5) $$F_{C,7}=41.84+1.895\times D-0.202\times A-1.411\times D\times A-0.514\times D^{2}-1.342\times A^{2}$$

(6) $$F_{C,28}=50.18+3.522\times D+2.407\times A-2.112\times D\times A-0.643\times D^{2}-1.948\times A^{2}$$

The terms *F_C_*_,7_, and *F_C_*_,28_ symbolize the 7-day, and 28-day compressive strengths, respectively, in MPa, and *D* and *A* refer to the DPF and PAC, respectively, in %.

The ANOVA summary for the compressive strengths of the DPFRC are highlighted in Table 6. The null hypothesis of no correlation connecting the responses and variables was tested and found to be false because all the models were highly significant as their P-scores were very small (0.0001). Consequently, based on the confidence interval (*p* < 0.05), the null hypothesis is rejected. Additionally, using their P-scores, i.e., *p* < 0.05, each model term’s significance was examined. Only the terms *D* and *A* were significant statistically for the 7-day compressive strength model having a P-score lower than 0.05; all remaining terms were non-significant. With regards to the 28-day strength model, the terms *D*, *A*, *D* × *A*, and *D*^2^ were found to have P-scores less than 0.05. The models for estimating the compressive strengths of the DPFRC were well correlated with their experimental data in terms of statistical significance. With respect to the lack of fits for all the compressive strength models, they were all non-significant compared to their respective pure errors.

Supplementary ANOVA validation was done to confirm the models’ performance, fitness, and validity utilizing the determination coefficient (R^2^) summary, as presented in Table 7. The R^2^ values for the 7- and 28-day compressive strength models are extremely high. For the 7- and 28-day compressive strength prototypes, their R^2^ scores of 0.901, and 0.925, respectively, clarified that only around 9.9%, and 7.5%, respectively, of the overall experimental results were not properly fitted to the models. The differences between the adjusted (modified) R^2^ and predicted (estimated) R^2^ values were further investigated. The discrepancy between the modified and estimated R^2^ values must be lower than 0.2 for a fitted model. The experimental data or the model ought to be inaccurate if the difference is bigger than 0.2. Outliers, model reduction, and response transformation are some ways to amend the model [36,37]. The disparity between the estimated and modified R^2^ scores for the 7-day compressive strength model was larger than 0.2. This indicated an issue with the model; thus, the model transformation was done. The estimated and modified R^2^ for the 7-day compressive strength responses agreed with each other after inverse transformation was applied to the models, as shown in Table 7. The estimated and modified R^2^ values for the 28-day compressive strength model were reasonably close to one another, with a difference of less than 0.2. All the models had relatively low standard deviations when compared to their corresponding mean values both prior and after transformation, which revealed that the experimental data have insignificant variability with the models. The transformed model equations for the 7-day compressive strength model are given by Equation (7).

(7) $$\frac{1}{F_{C,7}}=0.0253-0.0049\times D-0.0027\times A+0.0026\times D\times A+0.001\times D^{2}+0.0019\times A^{2}$$

#### 3.1.3. Analysis of Variance for Split Tensile Strength Models

Equations (8) and (9) represent the models generated to estimate the 7- and 28-day splitting tensile strength, respectively, for the DPFRC.

(8) $$F_{T,7}=2.682+0.211\times D+0.104\times A-0.047\times D\times A-0.07\times D^{2}-0.1\times A^{2}$$

(9) $$F_{T,28}=3.308+0.253\times D+0.145\times A-0.056\times D\times A-0.085\times D^{2}-0.127\times A^{2}$$

where F_T,7_ and F_T,28_ are the 7- and 28-day splitting tensile strengths, respectively, in MPa, A and D are the PAC and DPF in %

Table 8 depicts a summary of the ANOVA results for the proposed splitting tensile strength models. The models for the 7- and 28-day split-tensile strengths all have a *p* value less than 0.05. Therefore, according to the confidence interval, all the models were very significant. As a result, this explained that the null hypothesis that no connection existed between the parameters (PAC and DPF) and the response are bogus and rejected. Further significant tests were carried out on all the model terms to check for their significance in the respective models. In the models, the terms *D* and *A* represent DPF and PAC, respectively. Quadratic models were the most suitable and were selected for all the models. As a result, the model terms used in all the models for the splitting of the tensile strengths were *D*, *A*, *D*×*A*, *D*^2^, and *A*^2^. The terms *A* and *A*^2^ were the only significant terms in the 7- day splitting tensile strength equation. For the 28-day splitting tensile strength equation, only term *A* is significant and has a *p*-value under 0.05. The lack of fits for all the models were not significant compared to respective pure errors, which indicates how well the established models fitted the experimental data.

The results of additional statistical validations based on the degree of determination as calculated using the predicted residual error sum of squares (PRESS) are presented in Table 9. The R^2^ values for the 28-day splitting tensile strength model were good and over 0.84. The model with the best R^2^ value of 0.905 is the 7-day split tensile strength model, with only roughly 9.5% of the total experimental result could not be adequately fitted to the model. The discrepancy between the predicted and adjusted R^2^ values for the 7-day and 28-day splitting tensile strength models was more than 0.2. This indicated that there were issues with the experimental data or model. For this reason, it was not possible to utilize Equations (8) and (9) to estimate the 7- and 28-day splitting tensile strengths. Therefore, the necessary modification had to be made using either model reduction, response transformation, or outliers [36,37]. The 7- and 28-day split tensile strength models were transformed, and as seen in Table 9, their predicted and adjusted R^2^ scores agreed with each another as their difference was lower than 0.2. As a result, the transformed model shown in Equations (10) and (11) were the most suitable for predicting the 7- and 28-day split tensile strength of the DPFRC, respectively.

(10) $$\frac{1}{F_{T,7}}=0.38-0.038\times D-0.034\times A+0.011\times D\times A+0.012\times D^{2}+0.022\times A^{2}$$

(11) $$\frac{1}{F_{T,28}}=0.308-0.027\times D-0.028\times A+0.0073\times D\times A+0.0089\times D^{2}+0.018\times A^{2}$$

#### 3.1.4. Analysis of Variance for Flexural Strengths and Water Absorption

The model equations established for the prediction and estimation of the 7-day flexural strength, 28-day flexural strength, and water absorption of the DPFRC are given as Equations (12)–(14) respectively.

(12) $${Ɓ}_{7}=4.446+0.475\times D-0.013\times A-0.173\times D\times A-0.078\times D^{2}-0.072\times A^{2}$$

(13) $${Ɓ}_{28}=5.481+0.646\times D-0.07\times A-0.216\times D\times A-0.119\times D^{2}-0.067\times A^{2}$$

(14) $$W=3.197+0.386\times D-0.903\times A-0.0381\times D\times A-0.0035\times D^{2}+0.407\times A^{2}$$

For which Ɓ_7_ and Ɓ_28_ denote the 7- and 28-day flexural strengths, respectively, in MPa, *W* denotes the water absorption in %, and *D* and *A* represent DPF and PAC, respectively, in %.

Table 10 highlights a summary of the ANOVA for water absorption and the flexural strength models. With P-scores significantly lower than 0.05, all the models were statistically significant. Thus, the presumptive null hypothesis was untrue. Additionally, the confidence interval was employed to assess each model term’s significance (*p*-value 0.05). The only terms that were significant were *A* and *D* × *A* in the 7- and 28-day flexural strength models having P-scores below 0.05; the remaining terms were not significant. With reference to the water absorption model, *D*, *A*, and *A*^2^ were statistically significant with P-scores lower than 0.05. However, the terms *D* × *A* and *D*^2^ were non-significant with P-scores higher than 0.05 in the water absorption model. Regarding the lack of fits, the P-values for the water absorption and 7-day flexural strength models were both higher than 0.05, therefore their lack of fits were not statistically significant when compared to their corresponding pure errors. The 7-day flexural strength and water absorption models were thus considered to be reliable and well-fitting models. The 28-day flexural strength model, on the other hand, exhibited a significant lack of fit in comparison to its pure error. This indicates that the model is flawed and has poor fitting, and the issue may stem from the model or the data. Thus, model transformation was applied on the 28-day flexural strength model to address the significant lack of fit.

Utilizing the R^2^ ANOVA summary shown in Table 10, additional statistical validations and checks were carried out. The 7-day flexural strength and water absorption models had high R^2^ scores above 0.93. In contrast, the 28-day flexural strength’s R^2^ score of 0.852 indicated that around 14.8% of the entire experimental results did not suit the model accurately.

For the 7-day flexural strength and water absorption models, the discrepancy between their predicted and adjusted R^2^ values was below 0.2. The generated model equations (Equations (12) and (14)) could be utilized to estimate the responses without the requirement for any model reduction or transformation. The variation between the predicted and adjusted R^2^ scores for the 28-day flexural strength model was above 0.2. As a result, the derived equation in Equation (13) could not be used for prediction, indicating that there was an issue with the 28-day flexural strength model and/or the experimental results. To correct the significant lack of fit and the error of the variation of the adjusted and predicted R^2^ scores, model transformation and reduction were applied to the 28-day flexural strength model. The lack of fit for the 28-day flexural strength model became non-significant following the model’s inverse transformation and reduction as its P-score was greater than 0.05, as demonstrated in Table 10. The variance between the adjusted and predicted R^2^ for the 28-day flexural strength decreased to below 0.2 as depicted in Table 11. Equation (15) is a model equation that was developed to estimate the 28-day flexural strength of DPFRC after transformation.

(15) $$\frac{1}{F_{F,28}}=0.18-0.033\times D+0.01\times A+0.011\times D\times A+0.0059\times A^{2}$$

### 3.2. Diagnostic Plots for All the Models

**Figure 5:**
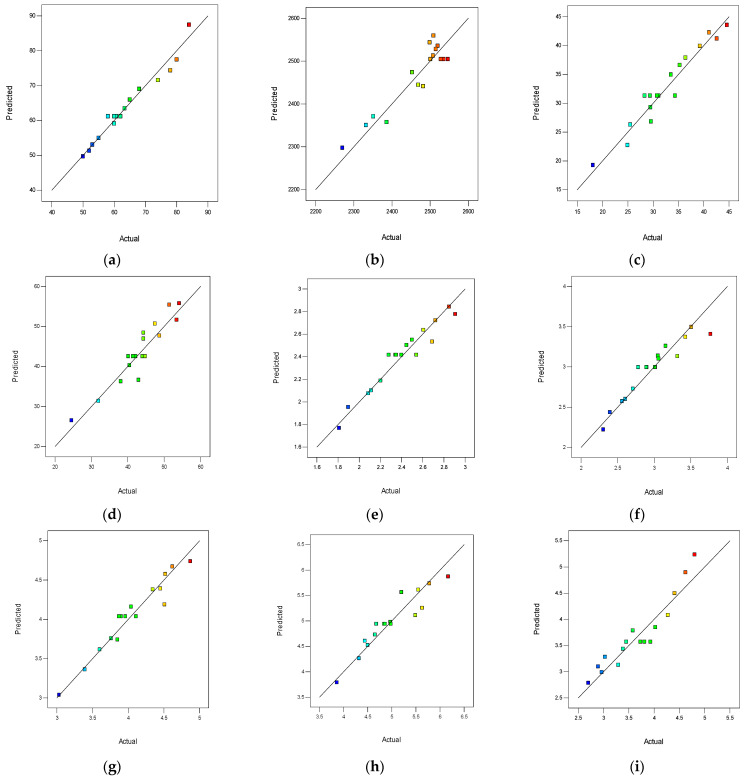
Predicted Versus Actual Plots. (**a**) Slump, (**b**) Fresh Density, (**c**) 7-day Compressive Strength, (**d**) 28-day Compressive Strength, (**e**) 7-day Split Tensile Strength, (**f**) 28-day Split Tensile Strength, (**g**) 7-day Flexural Strength, (**h**) 28-day Flexural Strength, (**i**) Water absorption.

Figure 5a–i presents the predicted versus actual plots for all the models. These plots were used to check and assess the correlation, fitness and predicting accuracy for all the models. A perfectly fitted model would have all its data points perfectly aligned across the straight trend line in its predicted versus actual plots. From Figure 5 presented, it can be seen that the data points for all the models were reasonably fitted to the straight trend line drawn. This justified the high degree of determination (R^2^) for all the models, which were all greater than 0.9 after transformation. The data points for the slump model in Figure 5a was the best fitted, which explained its highest R^2^ of 0.961 compared to all the other models. In Figure 5, the colors assigned to the data points explain the ranking of each of the responses. The blue colors represent the lowest responses, the green color represents the median responses, and the red colors represent the peak (highest) responses in the plots.

### 3.3. Influence of DPF and PAC on the Fresh Properties of DPFRC

**Figure 6:**
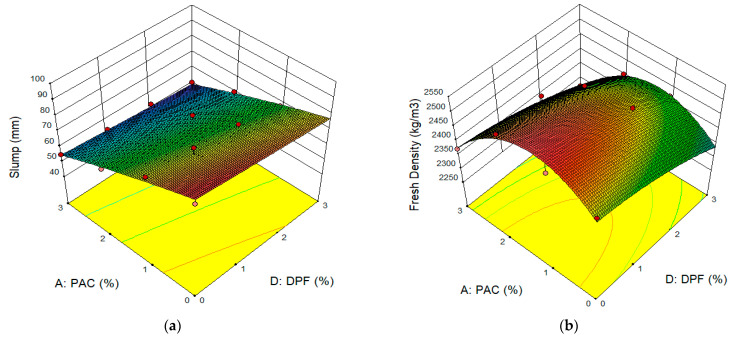
Surface Plots for Slump and Fresh Density. (**a**) Slump, (**b**) Fresh Density.

The 3D response surface plot for slump and fresh density is presented in Figure 6a,b, respectively. The effect of the hybrid of DPF and PAC on the workability of the concrete can be seen in Figure 6a. Mixes with low DPF content up to 2% and 0% to 1% PAC have the highest slump, whereas mixes with higher PAC content above 1% and more than 2% DPF have the lowest slump, as depicted by the green to blue regions in the 3D plots of Figure 6a. Therefore, it can be said that both DPF and PAC caused a reduction in the concrete’s slump. The reduction in slump due to DPF resulted from the high absorption-capacity of water for the DPF. The DPF, due to its hydrophilicity, porous structure, and amorphous nature of the cellulosic structure, led to the absorption of part of the mixing water in the fresh concrete, thereby resulting in reduced workability [4,15,48]. Furthermore, a further reduction in the slump was observed with an increase in the percentage addition of PAC. This is ascribed to the larger surface area of the PAC, giving it the ability to absorb part of the mixing water and reduce the workability [49]. Previous studies have also shown that PAC as an additive in cementitious composites led to a reduction in workability and fluidity of the cementitious composites [50].

From Figure 6b, the concrete’s fresh density somewhat decreased with the addition of DPF. This reduction in the density might be due to DPF’s water absorption, thus increasing the air content in the concrete. This leads to the escalation of pore volume and consequently a reduction in density [4]. The addition of up to 2% PAC resulted in an increase in the fresh density of the DPF concrete. The increase in density might be due to the PAC’s finer particles, thereby filling the pores within cement paste created by the DPF and densifying the concrete matrix. Additionally, since the PAC is used as an additive in the concrete, it increases the volume of the constituent materials and hence the unit weight of the concrete increases [35].

### 3.4. Influence of DPF and PAC on the Hardened Properties of DPFRC

#### 3.4.1. Compressive Strength of DPFRC

**Figure 7:**
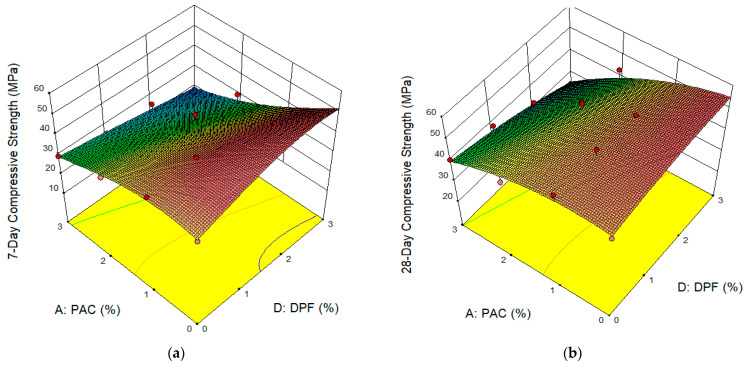
Surface Plots for Compressive Strengths. (**a**) 7-day Compressive Strength, (**b**) 28-day Compressive Strength.

Figure 7a,b displays the 3D surface response plot for the 7-day and 28-day compressive strengths of the DPFRC, respectively. These plots were used to discuss the influence of the hybrid of DPF and PAC on the compressive strength of the concrete. From the 3D plots, it can be observed that mixes containing the combinations of up to 3% DPF and 2% PAC yielded the highest compressive strength as represented by the reddish portions in the plots. This can be ascribed to the combined effects of the DPF and PAC, where the DPF’s capacity to make the concrete more ductile, which prevents cracks from forming and spreading. The DPF’s capacity to bridge cracks led to an increase in pre-cracking and post-cracking load resistance and improved the compressive load resistance even after the first crack had occurred before its ultimate failure [4,51]. The PAC played the role of filling the pores created by the DPF in the cement matrix, where it densified the microstructure and increased the concretes’ s strength [32,49]. The main cause of reduction in the concrete’s strength with the addition of DPF was the increase in pore volume [4], hence PAC, due to its filler ability, was able to mitigate this effect. However, the DPFRC containing the combination of 1% to 3% DPF and 3% PAC had the lowest compressive strengths at all ages as depicted by the green-to-blue color on the 3D plots. These lower strengths were caused by the carbon retardation and dilution effects of the PAC, which disrupted the hydration of tricalcium-aluminates [50]. However, from previous studies reported by several researchers, the compressive strength of cementitious composites, such as concrete and mortar, reduced with the increase in the addition of DPF, which they mainly attributed to the poor bonding between the fiber and the cement paste, an increase in pore volume in the cement matrix caused by the DPF [4,18,52]. With the addition of PAC to the DPFRC in this study, the loss in compressive strength due to the undesirable effects of the DPF on the cementitious composites were either fully or partially mitigated. This was due to the pore-filling effect and enhanced hydration reaction caused by the PAC in the concrete. This finding can be supported by the results of Rashad, et al. [50] who reported that up to 1.5% PAC as additive in cement paste caused significant improvement in compressive strength due to filler effect and escalation of hydration reaction. Lekkam, et al. [50] reported that up to 2% PAC as additive by weight of cement improved the strengths of cementitious composites.

#### 3.4.2. Split Tensile Strength of DPFRC

**Figure 8:**
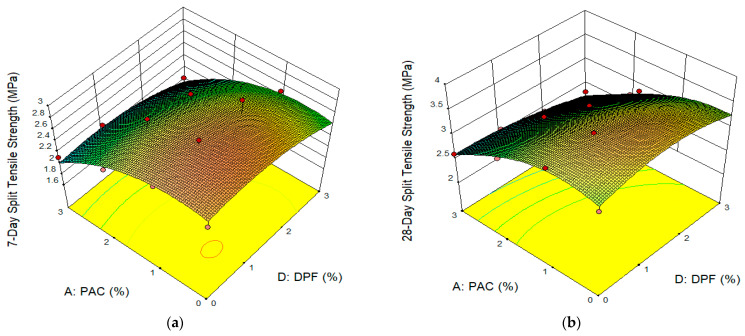
Surface Plots for splitting tensile strength. (**a**) 7-day Split Tensile Strength, (**b**) 28-day Split Tensile Strength.

As shown in Figure 8, the impacts of the PAC and DPF on the splitting tensile strength of DPFRC were displayed in the form of 3D-response surface plots. In Figure 8, the mixes containing a hybrid of up to 2% DPF and 2% PAC had the highest split tensile strength at both 7 and 28 days. The improvement in the tensile strength was attributed to the combined effects of both DPF and PAC. As already reported by previous studies, the addition of up to 2% DPF to concrete without any additive improved its tensile strength. The improvement was attributed to the DPF’s capacity to make the concrete more ductile, which prevents cracks from forming and spreading. The DPF’s capacity to bridge cracks led to an increase in tensile load resistance [4,51]. Similarly, the enhancement in tensile strength of the DPFRC with the addition of up to 2% is ascribed to the pore fill-up capability of the PAC, thus densifying the concrete’s microstructure and enhancing the compressive strength. The concrete’s splitting tensile strengths are directly correlated with its compressive strength [53], hence the improvement in splitting tensile strength. The mixes with higher DPF and PAC contents have the lowest splitting tensile strengths, as depicted by the green-to-blue regions in the plots, hence the addition of more than 2% DPF and 2% PAC resulted in a reduction in tensile strengths at all ages. The large surface area of the PAC and the high-water absorption rates of the DPF, which led to the absorption of some of the mixing water and decreased the workability of the paste, seem to be responsible for the mix’s poor consistency. The poor consistency led to balling effects of the DPF and honeycomb/voids formation in the cement matrix; this increased paths for premature cracking and failure, hence resulting in lower tensile strength. From previous studies, it had been reported that the addition of DPF improved the tensile strength of concrete, which was attributed to the crack-bridging effect of the DPF in the matrix [4,17]. Therefore, with the addition of PAC to the DPFRC, further improvement in the split tensile strength occurred due to the combined effect of DPF and PAC, as the PAC filled the pores in the cement matrix and enhanced the hydration reaction, thereby leading to an improvement in tensile strength.

#### 3.4.3. Flexural Strength of DPFRC

**Figure 9:**
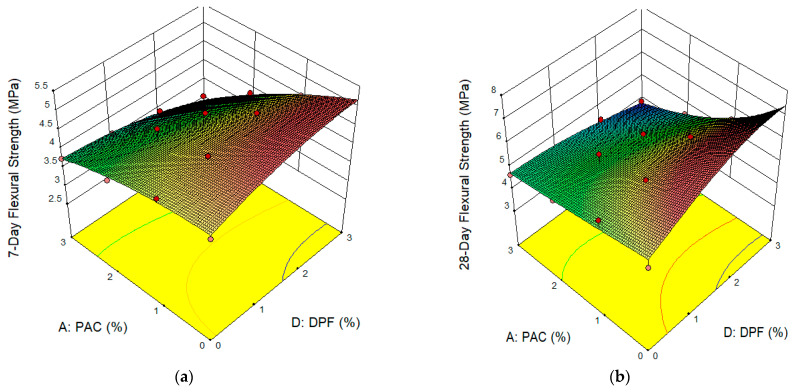
Surface Plots for flexural strengths. (**a**) 7-day Flexural Strength, (**b**) 28-day Flexural Strength.

The effect of the hybrid of DPF and PAC on the flexural strength of the DPFRC is presented in the form of a 3D response surface plot as presented in Figure 9. The reddish region on the 3D plots for 7- and 28-days’ flexural strengths represents the regions of highest flexural strengths. Therefore, the hybrid of up to 3% DPF with up to 2% PAC resulted in significant improvement in flexural strengths. The improvement in bending resistance (flexural strength) is due to the combined effects of DPF and PAC. For the DPF, due to its fibrous nature, its addition results in the enhancement of a crack-bridging effect of the concrete, leading to delays in the growth and propagation of the cracks with load application. This improves the bending resistance even after the first crack has occurred before its ultimate failure, hence enhancing the flexural strengths [4]. With regards to the PAC, its addition to the DPFRC densifies the concrete microstructure, resulting in improving the strength and bonding between the aggregate/fiber matrix and cement’s paste, consequently enhancing the flexural strengths. Previous studies have reported improvement in flexural strength with addition of DPF to cementitious composites which was attributed to the increase in ductile behavior and crack-bridging effect of the concrete with addition of DPF [4,54]. Similarly, Wang, et al. [32] reported that the addition of up to 2% PAC in mortar enhanced its flexural strength. From the 3D plots, the addition of 3% DPF and 3% PAC yielded the lowest flexural strengths at both 7 and 28 days, denoted by the bluish region in the plots. This might be due to the low workability caused by the high PAC contents, which led to more pores and honeycombs in the hardened matrix. This created a weak path for cracking to occur easily with load applications. This led to a reduction in the flexural strengths.

#### 3.4.4. Water Absorption of DPFRC

**Figure 10:**
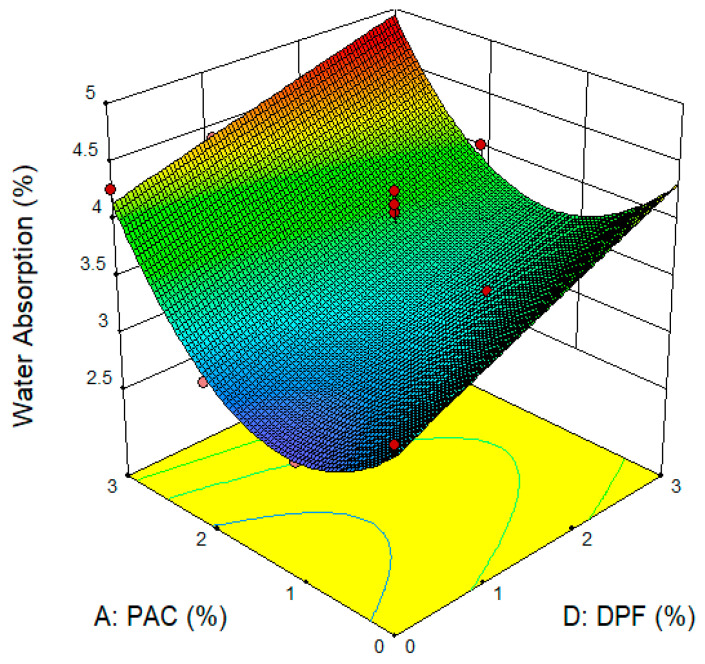
Surface Plots for Water Absorption.

The influence of DPF and PAC on the water absorption of DPFRC is shown in Figure 10. From the 3D plot, the lowest water absorption rate denoted by the blue region in the graph fell within the coordinates with 0% to 2% PAC and up to 2% DPF. Similar to previous studies [4], with the introduction of DPF, the water absorption escalated, resulting from the increased pore volume inside the concrete matrix. Another explanation could be that the DPF’s hydrophilic properties caused it to absorb additional water into the concrete [4]. The decline or reduction in the water absorption due to the addition of up to 2% PAC could be ascribed to the large surface area of the PAC which resulted in pore filling and densification of the concrete matrix, and hence a reduction in water absorption. Lekkam, et al. [50] reported that the addition of up to 2% activated carbon to cementitious materials resulted in densification of its microstructure due to the PAC’s filler effect. The addition of more than 2% PAC to the DPF containing 1% to 3% DPF led to an escalation in water absorption, as presented by the yellow-to-reddish regions of the 3D plot. This escalation in water absorption might be due to the undesirable effect of using higher dosage of PAC to the DPFRC. When a high quantity of PAC was added, a reduction in consistency occurred due to its large surface area. This caused improper dispersion of the paste and fibers in the cement matrix, causing high pore volume in the hardened cement matrix and consequently higher water absorption.

### 3.5. Multi-Objective Optimization

For the purpose of maximizing the workability and mechanical strengths and minimizing the water absorption of the DPFRC, a multi-objective optimization approach from the RSM package was used. In order to reach the correct proportions and potential combinations of the parameters to produce the desired results, the optimization was done by establishing some objectives (goals) for each of the variables and response. Table 12 summarizes the optimization criteria and objectives for the variables and responses, with the workability and mechanical strengths being maximized, and water absorption being minimized. The range for the variables, i.e., between 0% and 3% each, was maintained for the PAC and DPF during the optimization. 

The result of the optimization is presented in Table 12. From the results, the highest mechanical strength and workability (slump), and lowest water absorption was obtained by adding 0.93% DPF and 0.37% PAC by weight of cement and keeping all other constituent materials (as in Table 2) constant. The desirability is used to explain and validate how well and accurate was the optimization. A perfect optimization has a desirability of 100%, and a bad optimization has a very low desirability. The optimization possessed a high desirability of 91% according to Table 12.

### 3.6. Model Validations

The established models by RSM were validated using ANOVA. Further validation needs to be carried out through additional experiments. This will validate and ensure the practical applicability and accuracy of the models. The validation was done by estimating the responses using their respective final developed models (predicted results). Then, using a similar proportion of variables (PAC and DPF) utilized for calculating the predicted results, additional mixes were produced in the laboratory together with the other constant constituent materials. The mixes were then tested for fresh density, slump, compressive strengths, splitting tensile strengths, flexural strengths, and water absorption. The results were tagged experimental results. The models were then validated utilizing errors between the experimental and estimated results which was calculated using Equation (16).

(16) $$\Sigma =\frac{\chi -Ρ}{\chi }\times 100$$

In Equation (16), *Σ* represented the error in %, *χ* represents an experimental result, and *P* represents estimated result.

Table 13 provides a summary of the findings from the experimental validation of all the models. All the models had errors of less than 5.5%. Thus, using PAC and DPF as the variables, all the generated model equations can be employed to estimate the properties of DPFRC with high accuracy and fewer errors.

## 4. Conclusions

In this work, RSM was employed to examine how DPF and PAC as additives affected the properties of DPF-reinforced concrete (DPFRC) in both fresh and hardened states. The following findings resulted from the RSM analysis and experimental outcomes.

(1) Adding both PAC and DPF led to a reduction in workability (slump) of the DPFRC. Furthermore, DPF addition reduced the density of the concrete, whereas up to 2% PAC addition enhanced the concrete’s density.
(2) The combinations of 1 to 3% DPF with up to 2% PAC resulted in improvement in the compressive, split tensile and flexural strengths of the DPFRC. The combination of 1 to 3% DPF with 3% PAC yielded the lowest mechanical strengths.
(3) The DPFRC’s strengths were increased, and the amount of water absorption was minimized by adding 2 wt% of PAC.
(4) The models developed to estimate the slump, density, strength, and water absorption of DPFRC were highly significant with excellent correlations and predictive power. When experimentally validated, all the models exhibited average errors that were lower than 5.5%.
(5) From the multi-objective optimization results, the highest slump, compressive strength, flexural strength and split tensile strength and lowest water absorption rate were achieved using a combination of 0.93 wt% of DPF and 0.37 wt% of PAC as an additive. According to the results of the multi-objective optimization, the optimization’s outcome had a 91% desirability.

## Figures and Tables

**Table 1:** Chemical properties of binder materials [17].

Oxides	Compositions (%)	
	OPC	PAC
C	-	91
CaO	65.18	0.53
Al_2_O_3_	5.39	0.64
Fe_2_O_3_	3.4	0.60
SiO_2_	19.17	1.57
MgO	0.91	0.22
TiO_2_	0.24	0.06
Na_2_O	0.17	-
K_2_O	1.22	0.06
P_2_O_5_	0.09	0.15
SO_3_	3.51	1.31
MnO	0.18	-

**Table 2:** Physical properties of aggregates.

Properties	Fine Aggregate	Coarse Aggregate
Maximum Size (mm)	4.75	19
Specific gravity	2.63	2.67
Bulk density (kg/m^3^)	1565	1455
Water absorption (%)	1.87	0.65
Fineness modulus	2.26	−
Mud content (%)	1.1	−

**Table 3:** Mix Proportions.

Mix No.	Variables		Quantities for 1 kg/m^3^ (kg/m^3^)						
	DPF (%)	PAC (%)	Cement	DPF	PAC	Fine Aggregate	Coarse Aggregate	Water	S. P
M1	0	0	480	0.0	0.0	730	890	180	4.8
M2	0	1	480	0.0	4.8	730	890	180	4.8
M3	1	1	480	4.8	4.8	730	890	180	4.8
M4	2	1	480	9.7	4.8	730	890	180	4.8
M5	3	1	480	14.5	4.8	730	890	180	4.8
M6	0	2	480	0.0	9.6	730	890	180	4.9
M7	1	2	480	4.9	9.6	730	890	180	4.9
M8 *	2	2	480	9.8	9.6	730	890	180	4.9
M9	3	2	480	14.7	9.6	730	890	180	4.9
M10	0	3	480	0.0	14.4	730	890	180	4.9
M11	1	3	480	4.9	14.4	730	890	180	4.9
M12	2	3	480	9.9	14.4	730	890	180	4.9
M13	3	3	480	14.8	14.4	730	890	180	4.9

* Five times repeated to calculate lack of fitting relative to pure errors. S. P = Superplasticizer.

**Table 4:** Experimental Results.

Mix No.	Variables		Slump (mm)	Fresh Density (kg/m^3^)	Compressive Strength (MPa)		Splitting Tensile Strength (MPa)		Flexural Strength (MPa)		Water Absorption (%)
	DPF (%)	PAC (%)			7-Day	28-Day	7-Day	28-Day	7-Day	28-Day	
M1	0	0	84	2469	39.24	48.71	2.61	3.16	4.34	5.2	3.29
M2	0	1	80	2506	42.58	53.42	2.72	3.43	4.45	5.63	2.70
M3	1	1	78	2515	44.61	54.13	2.85	3.51	4.62	5.78	2.89
M4	2	1	74	2487	41.08	51.39	2.91	3.77	4.87	6.17	3.39
M5	3	1	68	2420	36.42	47.48	2.50	3.05	4.52	5.55	3.58
M6	0	2	65	2528	35.25	44.32	2.45	3.06	4.04	4.97	2.97
M7	1	2	63	2539	33.52	44.32	2.69	3.32	4.51	5.49	3.03
M8	2	2	61	2448	31.11	42.15	2.34	2.90	3.96	4.84	3.73
M9	3	2	60	2404	29.59	42.96	2.20	2.71	3.85	4.5	4.03
M10	0	3	55	2373	29.47	40.48	2.11	2.60	3.76	4.65	4.27
M11	1	3	53	2348	25.48	38.09	2.08	2.56	3.60	4.44	4.41
M12	2	3	52	2312	24.95	31.95	1.90	2.39	3.39	4.32	4.62
M13	3	3	50	2270	18.10	24.53	1.81	2.30	3.03	3.86	4.80
M8 *	2	3	60	2446	29.42	40.13	2.28	3.01	4.11	4.68	3.93
M8 *	2	3	60	2467	34.35	44.76	2.40	2.89	3.90	4.98	3.45
M8 *	2	3	62	2431	28.34	41.43	2.35	2.78	3.87	4.86	3.81
M8 *	2	3	58	2448	30.76	44.00	2.54	3.01	3.96	4.84	3.73

* M8 four times repeated to calculate lack of fitting relative to pure errors.

**Table 5:** Slump and fresh density models’ ANOVA.

Response	Source	F Value	*p*-Value Prob > F	Significance	R^2^	Adjusted R^2^	Predicted R^2^	A.P
Slump (mm)	Model	54.61	<0.0001	Yes	0.961	0.944	0.777	26.88
	D-DPF	14.04	0.0032	Yes				
	A-PAC	108.39	<0.0001	Yes				
	*D* × *A*	0.27	0.6131	No				
	*D* ^2^	0.00339	0.9546	No				
	*A* ^2^	0.26	0.6178	No				
	Lack of Fit	3.69	0.1123	No				
Fresh Density (kg/m^3^)	Model	39.54	<0.0001	Yes	0.95	0.923	0.852	22.35
	D-DPF	41.64	<0.0001	Yes				
	A-PAC	9.43	0.0106	Yes				
	*D* × *A*	0.00792	0.9307	No				
	*D* ^2^	2.71	0.1280	No				
	*A* ^2^	52.35	<0.0001	Yes				
	Lack of Fit	3.63	0.1152	No				

A.P = Adequate Precision.

**Table 6:** Compressive Strengths Models ANOVA.

Response	Source	Before Model Reduction			After Model Reduction		
		F Value	*p*-Value Prob > F	Significant	F Value	*p*-Value Prob > F	Significant
7-Day Compressive Strength (MPa)	Model	20.00	<0.0001	Yes	29.84	<0.0001	Yes
	D-DPF	5.51	0.0387	Yes	7.81	0.0174	Yes
	A-PAC	35.45	<0.0001	Yes	46.41	<0.0001	Yes
	*D* × *A*	3.48	0.0892	No	13.05	0.0041	Yes
	*D* ^2^	0.54	0.4774	No	2.27	0.1602	No
	*A* ^2^	2.01	0.1843	No	4.38	0.0604	Yes
	Lack of Fit	1.51	0.3634	No	1.24	0.4427	No
28-Day Compressive Strength (MPa)	Model	26.92	<0.0001	Yes			
	D-DPF	5.04	0.0462	Yes			
	A-PAC	44.03	<0.0001	Yes			
	*D* × *A*	8.93	0.0124	Yes			
	*D* ^2^	0.97	0.3456	No			
	*A* ^2^	4.84	0.0500	Yes			
	Lack of Fit	2.06	0.2532	No			

**Table 7:** Co-efficient of Regression ANOVA for Compressive Strength.

Factor	7-Day Compressive Strength (MPa)		28-Day Compressive Strength (Mpa)
	No Transformation	After Transformation	No Transformation
R^2^	0.901	0.931	0.925
Adjusted R^2^	0.856	0.900	0.890
Predicted R^2^	0.562	0.797	0.730
Adequate Precision	15.67	19.49	17.77
Standard Deviation	2.61	0.00249	2.44
Mean	32.60	0.032	43.19
C.V.%	8.01	7.74	5.65
PRESS	331.92	0.0002	234.47

PRESS = Predicted Residual Error Sum of Squares, C.V = Coefficient of Variation.

**Table 8:** Summary of ANOVA for Splitting Tensile Strength.

Responses	Sources	No Model Transformation			After Model Transformation		
		F Values	*p*-Values Prob > F	Significant	F Values	*p*-Values Prob > F	Significant
7-Day Splitting Tensile Strength (MPa)	Model	20.91	<0.0001	Yes	42.30	<0.0001	Yes
	D-DPF	4.26	0.0636	No	9.17	0.0115	Yes
	A-PAC	31.37	0.0002	Yes	57.02	<0.0001	Yes
	*D* × *A*	1.95	0.1905	No	5.68	0.0363	Yes
	*D* ^2^	5.07	0.0457	Yes	8.06	0.0161	Yes
	*A* ^2^	5.58	0.0377	Yes	14.86	0.0027	Yes
	Lack of Fit	1.63	0.3325	No	0.79	0.6303	No
28-Day Splitting Tensile Strength (MPa)	Model	12.22	0.0003	Yes	26.71	<0.0001	Yes
	D-DPF	2.51	0.1417	No	6.23	0.0297	Yes
	A-PAC	17.86	0.0014	Yes	34.54	0.0001	Yes
	*D* × *A*	1.07	0.3233	No	2.69	0.1292	No
	*D* ^2^	2.89	0.1174	No	4.74	0.0520	No
	*A* ^2^	3.55	0.0861	No	10.46	0.0079	Yes
	Lack of Fit	5.27	0.0636	No	2.24	0.2277	No

**Table 9:** Co-efficient of Regression ANOVA for split tensile strength.

Factors	7-Day Splitting Tensile Strength (MPa)		28-Day Splitting Tensile Strength (MPa)	
	No Transform	Model Transform	No Transform	Model Transform
R^2^	0.905	0.951	0.847	0.924
Adjusted R^2^	0.862	0.928	0.778	0.889
Predicted R^2^	0.657	0.884	0.309	0.728
Adequate Precision	15.96	22.88	12.17	17.99
Standard Deviation	0.12	0.02	0.19	0.015
Mean	2.40	0.42	2.97	0.34
C.V.%	4.85	3.70	6.27	4.47
PRESS	0.54	0.0064	1.73	0.0092

PRESS = Predicted Residual Error Sum of Squares, C.V. = Coefficient of Variation.

**Table 10:** ANOVA’s Summary of water absorption and flexural strength models.

Responses	Sources	Before Model Transformation			After Model Transformation		
		F Values	*p*-Values Prob > F	Significant	F Values	*p*-Values Prob > F	Significant
7-Day Flexural Strength (MPa)	Model	23.67	<0.0001	Yes	-	-	-
	D-DPF	0.18	0.6754	No	-	-	-
	A-PAC	52.12	<0.0001	Yes	-	-	-
	*D* × *A*	13.06	0.0041	Yes	-	-	-
	*D* ^2^	3.12	0.1049	No	-	-	-
	*A* ^2^	1.42	0.2587	No	-	-	-
	Lack of Fit	4.48	0.0830	No	-	-	-
28-Day Flexural Strength (MPa)	Model	12.67	0.0003	Yes	29.64	<0.0001	Yes
	D-DPF	0.20	0.6651	No	0.38	0.5506	No
	A-PAC	28.41	0.0002	Yes	96.95	<0.0001	No
	*D* × *A*	7.43	0.0197	Yes	24.01	0.0004	No
	*D* ^2^	2.63	0.1331	No	7.49	0.0181	No
	*A* ^2^	0.45	0.5140	No	-	-	
	Lack of Fit	9.77	0.0219	Yes	4.63	0.0779	No
Water Absorption (%)	Model	33.93	<0.0001	Yes	-	-	-
	D-DPF	36.56	<0.0001	Yes	-	-	-
	A-PAC	12.35	0.0049	Yes	-	-	-
	*D* × *A*	0.52	0.4871	No	-	-	-
	*D* ^2^	0.00508	0.9444	No	-	-	-
	*A* ^2^	37.71	<0.0001	Yes	-	-	-
	Lack of Fit	1.11	0.4865	No	-	-	-

**Table 11:** Co-efficient of Regression ANOVA for flexural strength and water absorption models.

Factors	7-Day Flexural Strength (MPa)	28-Day Flexural Strength (MPa)		Water Absorption (%)
		No Transform	Model Transform	
R^2^	0.915	0.852	0.908	0.939
Adjusted R^2^	0.876	0.785	0.878	0.911
Predicted R^2^	0.753	0.235	0.740	0.804
Adequate Precision	17.57	13.14	20.30	20.57
Standard Deviation	0.17	0.27	0.0085	0.18
Mean	4.05	4.99	0.20	3.68
C.V.%	4.10	5.49	4.18	4.97
PRESS	0.88	4.27	0.0025	1.19

PRESS = Predicted Residual Error Sum of Squares, C.V = Coefficient of Variation.

**Table 12:** Goals and results of multi-Objective optimization.

Names	Units	Goals	Lower Limit	Upper Limit	Solution
A: DPF	%	In range	0	3	0.93
A: PAC	%	In range	0	3	0.37
Slump	mm	Maximize	50	84	80
Fresh Density	Kg/m^3^	In range	2270	2539	2482
7-Day Compressive Strength	MPa	Maximize	18.10	44.61	46.55
28-Day Compressive Strength	MPa	Maximize	24.53	54.13	52.80
7-Day Split Tensile Strength	MPa	Maximize	1.81	2.91	2.80
28-Day Split Tensile Strength	MPa	Maximize	2.30	3.77	3.52
7-Day Flexural Strength	MPa	Maximize	3.03	4.87	4.75
28-Day Flexural Strength	MPa	Maximize	3.86	6.17	6.17
Water Absorption	%	Minimize	2.70	4.80	3.26
Desirability	%	-	-	-	0.91

**Table 13:** Goals and results of multi-objective optimization.

Responses	Variables (%)		Predicted		Experimental		Errors (%)		Average Error (%)	
	DPF	PAC								
Slump (mm)	0.93	0.37	80.5		84		4.21		4.97	
	2	2	61.6		65		5.29			
	1.5	0.5	77.6		75		3.42			
Fresh Density (kg/m^3^)	0.93	0.37	2482		2411		2.94		3.41	
	2	2	2454		2336		5.07			
	1.5	0.5	2474		2376		4.13			
Water absorption (%)	0.93	0.37	3.26		3.11		4.87		5.34	
	2	2	3.62		3.85		5.85			
	1.5	0.5	3.39		3.22		5.27			
Compressive Strength (MPa)	**DPF**	**PAC**	**7D**	**28D**	**7D**	**28D**	**7D**	**28D**	**7D**	**28D**
	0.93	0.37	45.95	52.80	43.76	50.41	6.71	5.00	5.17	3.94
	2	2	31.15	43.23	32.16	45.17	3.74	3.13		
	1.5	0.5	47.00	53.15	44.74	51.26	5.06	3.68		
Split Tensile Strength (MPa)	0.93	0.37	2.86	3.51	2.98	3.70	3.92	5.24	3.93	4.56
	2	2	2.40	2.99	2.29	2.87	4.97	4.07		
	1.5	0.5	2.88	3.53	2.97	3.38	2.90	4.36		
Flexural Strength (MPa)	0.93	0.37	4.83	6.35	5.15	6.63	4.30	4.87	4.56	5.33
	2	2	4.03	4.96	4.31	5.29	6.23	5.85		
	1.5	0.5	4.96	6.89	5.27	7.11	3.15	5.27		

## Data Availability

Data will be available on request.
